# Multimorbidity and achievement of treatment goals among patients with type 2 diabetes: a primary care, real-world study

**DOI:** 10.1186/s12913-021-06989-x

**Published:** 2021-09-14

**Authors:** Eveliina Heikkala, Ilona Mikkola, Jari Jokelainen, Markku Timonen, Maria Hagnäs

**Affiliations:** 1 Rovaniemi Health Center, Koskikatu 25, 96200 Rovaniemi, Finland; 2 Medical Research Center Oulu, University of Oulu and Oulu University Hospital, PO Box 5000, 90014 Oulu, Finland; 3 Center for Life Course Health Research, University of Oulu, PO Box 5000, 90015 Oulu, Finland

## Abstract

**Background:**

Type 2 diabetes (T2D), with its prevalence and disability-causing nature, is a challenge for primary health care. Most patients with T2D are multimorbid, i.e. have one or more long-term diseases in addition to T2D. Multimorbidity may play a role in the achievement of T2D treatment targets, but is still not fully understood. The aims of the present cross-sectional, register-based study were to evaluate the prevalence and the most common patterns of multimorbidity among patients with T2D; and to study the potential associations between multimorbidity and treatment goal achievement, including measurements of glycosylated haemoglobin A1c (HbA1c), low-density lipoprotein (LDL) and systolic blood pressure (sBP).

**Methods:**

The study population consisted of 4545 primary care patients who received a T2D diagnosis between January 2011 and July 2019 in Rovaniemi Health Centre, Finland. Data on seven long-term concordant (T2D-related) diseases, eight long-term discordant (non-T2D-related) diseases, potential confounders (age, sex, body mass index, prescribed medication), and the outcomes studied were collected from patients’ records. Logistic regression models with odds ratios (ORs) and 95 % confidence intervals (CIs) were assessed to determine the associations between multimorbidity and the achievement of treatment targets.

**Results:**

Altogether, 93 % of the patients had one or more diseases in addition to T2D, i.e. were considered multimorbid. Furthermore, 21 % had only concordant disease(s) (Concordant subgroup), 8 % had only discordant disease(s) (Discordant subgroup) and 64 % had both (Concordant and discordant subgroup). As either single diseases or in combination with others, hypertension, musculoskeletal (MS) disease and hyperlipidaemia were the most prevalent multimorbidity patterns. Being multimorbid in general (OR 1.32, CI 1.01–1.70) and belonging to the Concordant (OR 1.45, CI 1.08–1.95) and Concordant and discordant (OR 1.31, CI 1.00–1.72) subgroups was associated with achievement of the HbA1c treatment target. Belonging to the Concordant and discordant subgroup was related to meeting the LDL treatment target (OR 1.31, CI 1.00–1.72).

**Conclusions:**

Multimorbidity, including cardiovascular risk and the musculoskeletal disease burden, was extremely prevalent among the T2D patients who consulted primary health care. Primary care clinicians should survey the possible co-existence of long-term diseases among T2D patients to help maintain adequate treatment of T2D.

## Background

Type 2 diabetes (T2D) is one of the most common and disability-causing long-term diseases worldwide [1]. Approximately 8 % of the global population is estimated to suffer from T2D during their lifetime [2]. It is predicted that between 2010 and 2030, the number of people living with diabetes in developed countries will increase by 20 % [3]. T2D is a metabolic disorder, which is associated with lifestyle and genetic factors [2, 4–6].

T2D rarely occurs without coexisting diseases. It has been reported that 77–90 % of patients with T2D also have one or more other diseases and can therefore be categorised as multimorbid [7, 8]. Typical diseases that coexist with T2D are hypertension and cardiovascular diseases [8, 9], but different pains and mental health problems are also prevalent [7–9]. The presence of coexisting diseases associates with patients’ unbeneficial self-care behaviours [10], macrovascular complications, hospitalisation, and mortality among patients with T2D [11, 12]. A significant part of T2D medical costs relate to complications and coexisting diseases [13].

In the prevention of T2D-related complications, it is essential that the treatment goals related to glucose, cholesterol, and blood pressure (BP) are achieved [14]. Despite the heightened risk of complications and mortality related to multimorbidity among patients with T2D [11, 12], the relevance of multimorbidity for achieving the goals of these clinical measures is not yet fully understood. Studies examining differentiating concordant (T2D-related) and discordant (non-T2D-related) diseases in multimorbidity patterns, as suggested by Piette & Kerr [15], have observed that concordant conditions positively affect the achievement of measured treatment goals [16–19], whereas the relationships between discordant diseases and goal achievement seem to be more conflicting [16, 18, 19]. In a recent systematic review, ten of the 14 included studies found no associations between multimorbidity and glycosylated haemoglobin A1c (HbA1c), whereas four of the 14 studies related multimorbidity to higher levels of HbA1c [20]. However, not all studies have observed an association between concordant conditions and lower HbA1c [9]. Only a limited number of studies have explored the role of multimorbidity in the achievement of treatment goals using a number of diabetic treatment measurements (HbA1c, low-density-lipoprotein cholesterol (LDL), and BP). Studies conducted in a primary care setting [20], focusing only on patients with T2D are especially lacking [16–19].

 To increase knowledge about multimorbidity among patients with T2D, we studied Northern Finnish T2D patients who consulted primary health care centres between 2011 and 2019. Our aims were to estimate the prevalence of multimorbidity; to evaluate which multimorbidity patterns are the most prevalent; and to assess whether multimorbidity is associated with the achievement of T2D treatment goals in a cross-sectional, real-world setting among Northern Finnish patients with T2D. We hypothesised that multimorbidity is extremely prevalent and that concordant diseases are positively, and discordant diseases negatively associated with achieving treatment goals for HbA1c, LDL, and systolic BP (sBP).

## Methods

### Study population and study protocol

The study population included primary care patients who lived in the city of Rovaniemi, Finland and who had received a T2D diagnosis between January 2011 and July 2019. T2D diagnosis was based on the ICD-10 (10th revision of the World Health Organization’s International Classification of Disease) codes E11.1–E11.9, which we found in the patient records of Rovaniemi’s health centre. During the time of data collection, altogether 4545 people were recorded as having T2D. Patients with no visits to primary care between 2017 and 2019 were excluded from the analysis to ensure that patients still lived in Rovaniemi and were alive. In Rovaniemi, all patients with T2D who are treated at the health centre are regularly called for planned T2D consultations at an interval of a maximum of one and a half years. The sample used in the cross-sectional analyses consisted of the number of observations with a corresponding outcome variable; for example, N for associations between multimorbidity and HbA1c was 4480.

Rovaniemi is a city in Northern Finland with approximately 62 000 people living in both urban and rural areas. All collected personal data were pseudonymised by replacing identifiable information with ID codes and were gathered anonymously. As this was a register-based study, according to contemporary Finnish legislation, no written consent was required from the participants. The study protocol was approved by the Ethics Committee of the Lapland Central Hospital (05/2018).

### Definition of multimorbidity

In the present study, multimorbidity was defined as the presence of one or more long-term disease in addition to T2D [21, 22]. Classification of long-term diseases as concordant and discordant diseases is presented in Table 1. Concordant diseases, which are thought to share a similar pathophysiology with T2D, were selected in line with earlier publications [7, 18]. To study cardiovascular diseases in general, we combined coronary heart disease, peripheral vascular disease, and stroke/TIA with cardiovascular diseases. The selection of discordant diseases, i.e. those not directly related to T2D, was based on their prevalence being 5 % or over in the present study population, which is a commonly applied method to assign long-term diseases in multimorbidity studies [23]. Musculoskeletal (MS) diseases included osteoarthritis, back diseases, inflammatory polyarthropathies, and soft tissue diseases, as these were the most prevalent in this category (prevalence rate > 5 % in the study sample) and are considered to be long term. Data on long-term diseases were collected from patient records using the corresponding ICD-10 codes.

**Table 1:** Classification of long-term diseases

**Disease(s)**	
Concordant diseases	
Hypertension	
Hyperlipidaemia	
Atrial Fibrillation	
Heart Failure	
Obesity	
Chronic kidney disease	
Cardiovascular diseases	
Discordant diseases	
Cancer	
Musculoskeletal diseases	
Asthma/chronic obstructive pulmonary disease	
Hyperplasia of the prostate	
Hypothyroidism	
Dementia/Alzheimer’s disease	
Depression	
Sleep disorders	

### Outcomes

The outcomes of the present study were the achievement of the below-mentioned treatment goals for HbA1c, LDL and sBP, which were dichotomised as yes vs. no. The latest values of all of these biochemical variables were applied and retrieved from patient records. Before a planned T2D consultation or during another BP check-up, patients were asked to measure their BP at home twice a day, twice per session, for 4 days [24]. The average of all sBP values was used. In the case of missing home measurements, we utilised the measurement data collected during the healthcare visit.

The targets of the above-mentioned biochemical variables were: sBP < 135 mmHg, HbA1c < 53 mmol/mol, and LDL < 2.5 mmol/L, in accordance with the latest Finnish national guidelines for the treatment of T2D [25]. As sBP values were monitored at home, the target was < 135 mmHg instead of < 140 mmHg as suggested in the national BP guideline [26].

### Covariates and variables used to characterise study population

The analyses of the present study were adjusted for age, sex, body mass index (BMI) and prescribed medications, collected from patient records. Participants reported their weight and height in a document sent before a planned T2D consultation or in a BP check-up. BMI (kg/m^2^) was divided according to the World Health Organization’s classification [27] as underweight/normal weight (BMI < 25.0), overweight (25.0–29.9), obesity (BMI 30.0 or over), and unknown. Data on prescriptions of antihyperglycemic, antihypertensive, and lipid lowering medications were gathered 2 years before and after the latest value of a corresponding clinical value, i.e. prescription of antihyperglycemic medication 2 years before and after the latest HbA1c value. The medications included, according to the ATC (Anatomical Therapeutic Chemical) codes, were: A10B, oral diabetes medications; A10A, insulins; C09A, angiotensin-converting enzyme inhibitors; C09C, angiotensin receptor blockers; C07AB, beta blockers; C08CA, calcium blockers; C03, diuretics; C10AA, statins; and C10AX, ezetimibe. Estimated glomerular filtration rate (eGFR) was assessed as a biochemical measure of renal function and was also obtained from patient records.

### Statistical methods

We regarded multimorbidity and its subgroups (Concordant, Discordant, and Concordant and discordant) as explanatory factors and the achievement of the treatment targets for HbA1c, LDL, and sBP as outcome factors. We used logistic regression analyses to test the potential associations between explanatory factors and outcome factors among patients with T2D, with odds ratios (ORs) and 95 % confidence intervals (CIs). We adjusted Model 1 for age, sex, and prescribed medications, and Model 2 for BMI in addition to covariates of Model 1. We calculated mean and standard deviation (SD) for age and the biochemical variables used, and the categorical variables were presented as proportions. Mean differences between multimorbidity groups for continuous variables were tested by independent samples t-test and analysis of variance; and by Pearson Chi-square test for categorical variables. Statistical analyses used IBM SPSS Statistics for Macintosh, Version 24.0. Armonk, NY: IBM Corp. IBM Corp. Released 2016. A *p*-value < 0.05 was considered statistically significant.

## Results

### Characterisation of studied patients with T2D

Of the 4545 patients with T2D, 46 % were women (Table 2). The mean age of the participants was 70.9 (12.3 SD), and their mean BMI was 29.8 (6.03 SD) kg/m^2^. The treatment goals for HbA1c, sBP, and LDL were achieved by 71 %, 46 %, and 56 % of patients with T2D, respectively. Almost all had been prescribed some antihyperglycemic medication (97 %), and over 75 % of the patients had also been prescribed antihypertensive, or lipid lowering medication of some kind.

**Table 2:** Characteristics of patients diagnosed with type 2 diabetes

	Values/percentages	N of observations
Sex, female, %	46	4545
Age, mean (SD)	70.9 (12.3)	4545
Body mass index, kg/m^2^, mean (SD)	29.8 (6.03)	3434
Underweight/normal weight, %	17	781
Overweight, %	29	1296
Obesity, %	30	1357
Unknown	24	1111
HbA1c, mmol/mol, mean (SD)	49.5 (13.4)	4480
sBP, mmHg, mean (SD)	138 (20.4)	3394
LDL, mmol/l, mean (SD)	2.52 (1.00)	4423
eGFR, ml/min/1.73m^2^, mean (SD)	78.2 (20.4)	4049
Proportion of patients who achieved treatment target for		
HbA1c (< 53 mmol/mol), %	71	4480
sBP (< 135 mmHg), %	46	3394
LDL (< 2.5 mmol/l), %	56	4423
Antihyperglycemic medication of any kind, %	97	4545
Antihypertensive medication of any kind, %	75	4545
Lipid lowering medication of any kind, %	88	4545

*HbA1c* glycosylated haemoglobin A1c, *sBP* systolic blood pressure, *LDL* low-density-lipoprotein cholesterol, *eGFR* estimated glomerular filtration rate, *SD* standard deviation

### Prevalence of multimorbidity and characteristics of patients in multimorbidity groups

The prevalence of multimorbidity was as high as 93 % (Table 3). Of the multimorbidity subgroups, the most common group was Concordant and discordant (64 %), followed by Concordant (21 %) and Discordant (8 %). The number of diseases in addition to T2D was mainly between one and two across the subgroups, except in the Concordant and discordant subgroup (3; mean 3.89). Among the multimorbid patients, the mean number of diseases was 3.23. Those without multimorbidity were slightly younger than their multimorbid counterparts (mean 62.6 vs. 71.5 years, *p* < 0.001). However, it is of note that the individuals without multimorbidity achieved the recommended levels of HbA1c (65 % vs. 71 %, *p* = 0.023) and LDL (40 % vs. 57 %, *p* < 0.001) less often than those with multimorbidity. We observed no significant differences between the age or sex of those with BMI data and those without, but slightly fewer patients without BMI data had achieved the HbA1c target (data not shown).

**Table 3:** Prevalence and mean values of demographics in multimorbidity and type 2 diabetes (T2D) only groups

	Multimorbidity	Concordant	Discordant	Concordant and discordant	T2D only
% (n) of study population	93 (4239)	21 (954)	8 (373)	64 (2912)	7 (306)
Sex, female, % (n)	46 (1960)	39 (372)	53 (197)	48 (1391)	41 (124)
Age, mean (SD)	71.5 (12.0)	68.9 (11.7)	65.6 (14.6)	73.1 (11.3)	62.6 (12.9)
Body mass index, kg/m^2^, mean (SD)	29.8 (6.06)	29.4 (5.78)	29.3 (6.06)	30.0 (6.13)	29.9 (5.45)
Under-/normal weight, % (n)	18 (747)	17 (159)	16 (58)	18 (530)	11 (34)
Overweight, % (n)	29 (1237)	28 (266)	26 (98)	30 (873)	19 (59)
Obesity, % (n)	30 (1291)	24 (233)	26 (95)	33 (963)	22 (66)
Unknown, % (n)	23 (964)	31 (296)	33 (122)	19 (546)	48 (147)
Proportion of patients who achieved treatment target for					
HbA1c, % (n)	71 (2965)	73 (682)	67 (242)	71 (2041)	65 (193)
sBP, % (n)	46 (1490)	47 (290)	50 (130)	45 (1070)	50 (78)
LDL, % (n)	57 (2372)	56 (525)	46 (161)	59 (1686)	40 (115)
Number of diseases, % (n)					
0					100 (308)
1	14 (606)	36 (345)	70 (261)		
2	21 (881)	42 (396)	23 (84)	14 (401)	
3	25 (1048)	18 (169)	6 (21)	30 (858)	
4	20 (852)	4 (40)	2 (6)	28 (806)	
5	12 (503)	0.5 (4)	0.5 (1)	17 (498)	
6	6 (234)			8 (234)	
7	2 (89)			3 (89)	
8	0.5 (19)			1 (19)	
9	0.2 (7)			0.5 (7)	
Mean number of diseases (SD)	3.23 (1.55)	1.91 (0.86)	1.40 (0.69)	3.89 (1.33)	

Concordant includes T2D-related diseases, Discordant includes non-T2D-related diseases, and Concordant and discordant includes both

Multimorbidity = one or more diseases in addition to T2D

*HbA1c* glycosylated haemoglobin A1c, *sBP* systolic blood pressure, *LDL* low-density-lipoprotein cholesterol, *SD* standard deviation

### Multimorbidity patterns

**Fig. 1:**
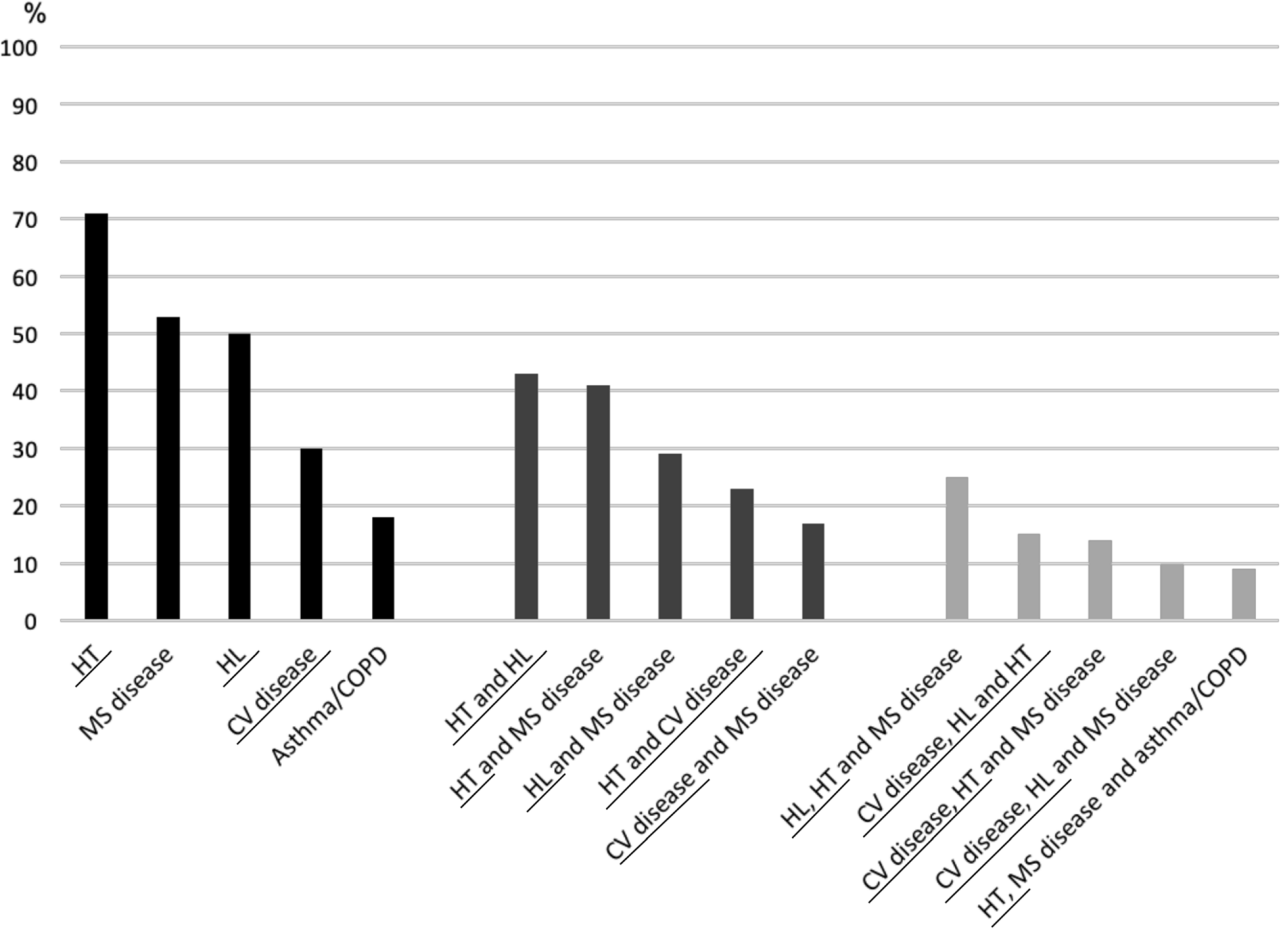
Five most common patterns of one, two and three additional diseases among the study population. *N* = 4545. HT = hypertension; MS = musculoskeletal; HL = hyperlipidaemia; CV = cardiovascular; COPD = chronic obstructive pulmonary disease. Concordant (type 2 diabetes-related) diseases are underlined

Figure 1 presents the most common disease patterns, including one to three diseases in addition to T2D among the study population (*n* = 4545). Patients with T2D most commonly suffered from hypertension (71 %), an MS disease (52 %) or hyperlipidaemia (50 %). Over 40 % of patients had hypertension combined with hyperlipidaemia or an MS disease, and nearly 30 % were affected by hyperlipidaemia and an MS disease. One-quarter of the patients were diagnosed with all the following three diseases: hypertension, hyperlipidaemia and an MS disease. Depression was uncommon (6 %, data not shown).

### Associations between multimorbidity and T2D treatment targets

Table 4 shows the ORs and CIs for the associations of multimorbidity and its subgroups with the achievement of the treatment goals for HbA1c, LDL and sBP. Being multimorbid and belonging to the Concordant and Concordant and discordant subgroups was significantly associated with the achievement of the HbA1c target in the fully adjusted model (OR 1.32, CI 1.01–1.70; OR 1.47, CI 1.10–1.95; OR 1.32, CI 1.01–1.72, respectively) and with the attainment of the LDL target in the model adjusted for age, sex and prescribed medications (OR 1.34, CI 1.03–1.74; OR 1.33, CI 1.00–1.78; OR 1.36, CI 1.05–1.78, respectively). However, in the Concordant and discordant subgroup, only the association with meeting the LDL treatment target remained statistically significant when BMI was taken into account (OR 1.31, CI 1.00–1.72). Belonging to the Discordant subgroup did not relate to any outcome in any model, and we observed no associations between multimorbidity or its subgroups and the achievement of the sBP treatment goal.

**Table 4:** Logistic regression analysis for associations between multimorbidity groups and achievement of T2D treatment targets

	HbA1c OR (95 % CI)	LDL OR (95 % CI)	sBP OR (95 % CI)	HbA1c OR (95 % CI)	LDL OR (95 % CI)	sBP OR (95 % CI)	HbA1c OR (95 % CI)	LDL OR (95 % CI)	sBP OR (95 % CI)
	Unadjusted			Model 1^a^			Model 2^b^		
Multimorbidity	**1.34** (1.04–1.71)	**2.00** (1.57–2.56)	0.84 (0.61–1.16)	**1.47** (1.14–1.89)	**1.34** (1.03–1.74)	0.82 (0.59–1.14)	**1.32** (1.01–1.70)	1.29 (0.99–1.68)	0.85 (0.61–1.18)
Concordant	**1.45** (1.09–1.90)	**1.91** (1.46–2.51)	0.86 (0.60–1.22)	**1.59** (1.20–2.11)	**1.33** (1.00–1.78)	0.82 (0.57–1.18)	**1.47** (1.10–1.95)	1.30 (0.98–1.74)	0.84 (0.58–1.20)
Discordant	1.12 (0.81–1.54)	1.25 (0.91–1.72)	0.98 (0.66–1.46)	1.13 (0.81–1.56)	1.22 (0.87–1.70)	0.98 (0.65–1.47)	1.03 (0.74–1.43)	1.19 (0.85–1.66)	0.98 (0.66–1.47)
Concordant and discordant	**1.34** (1.04–1.71)	**2.15** (1.68–2.76)	0.82 (0.59–1.14)	**1.50** (1.15–1.94)	**1.36** (1.05–1.78)	0.80 (0.57–1.11)	**1.32** (1.01–1.72)	**1.31** (1.00–1.72)	0.83 (0.59–1.16)
T2D only	1.0	1.0	1.0	1.0	1.0	1.0	1.0	1.0	1.0

Concordant includes T2D-related diseases, Discordant includes non-T2D-related diseases, and Concordant and discordant includes both

Multimorbidity = one or more diseases in addition to T2D

Significant ORs are bolded

*T2D * type 2 diabetes, *OR * odds ratio, *CI * confidence interval, *HbA1c * glycosylated haemoglobin A1c, *LDL * low-density-lipoprotein cholesterol, *sBP* systolic blood pressure

^a^Adjusted for: age, sex, prescribed medications

^b^Adjusted for: age, sex, prescribed medications, BMI

## Discussion

In this primary care, real-world study of 4545 patients with T2D, we observed that 93 % had one or more additional diseases. The majority of the multimorbid patients had both concordant and discordant diseases. Of the concordant diseases, hypertension, and of the discordant diseases, MS diseases were the most commonly coexisting diseases, appearing as individual diseases or in combination with other (mainly concordant) diseases among the study population. Surprisingly, depression was not included in the most frequent multimorbidity patterns. Being multimorbid in general and having only concordant disease(s) or a combination of concordant and discordant diseases was associated with the achievement of the HbA1c treatment target. Having both concordant and discordant diseases was also related to the attainment of the LDL treatment target.

In the current study, almost all the T2D patients had one or more diseases in addition to T2D. This is in line with the prevalence rate reported among Australian primary care patients (90 %; [7]), but slightly higher than the rates among English (77 %; [8]), Croatian (78 %; [28]) and Danish patients (80 %; [29]). We found no studies assessing the frequency of multimorbidity by evaluating several diseases among Scandinavian patients with T2D other than that of Pouplier et al. [29]. Typically, the differences in the prevalence rates affiliate with the different definitions of multimorbidity used, but these studies (except [28]) defined multimorbidity as having one or more diseases in addition to T2D, as determined in the current study. Thus, discrepancies in the estimates could be due to different disease patterns being included in the analyses. Most of our patients had diagnoses of both concordant and discordant diseases, and having only concordant disease(s) was more common than having only discordant disease(s). Findings have been published that are both similar [19] and contradictory [7, 30].

MS diseases were highlighted in all the multimorbidity patterns with different numbers of additional diseases among the study sample. Congruently, Chiang et al. [7] found “painful conditions” to co-exist with T2D at a prevalence rate of 55 %, and Zghebi et al. [8] found osteoarthritis to be the third most common co-existing disease, with nearly one-fifth of T2D patients suffering from it. Being affected by an MS disease is likely to lead to a substantial disability burden and lower quality of life [31]. Along with its high prevalence, this emphasises the need to focus on possible MS disease symptoms among patients with T2D who consult primary care clinics. The generally high prevalence of MS diseases might explain its presence among T2D patients. On the other hand, a recent meta-analysis has shown that people with diabetes are more likely than those without diabetes to report different MS pains [32]. The co-existence of these two diseases might be accounted for by some metabolic/inflammatory and biomechanical processes [33, 34] and/or shared predisposing factors, such as obesity [35]. Our multimorbid patients did not significantly differ from the patients with T2D only with respect to BMI.

Surprisingly, depression was not presented in any of the most common multimorbidity patterns among the patients with T2D in the present study. This finding may be related to the low prevalence of depression, as only 6 % of the participants were diagnosed with it (data not shown). In a recent systematic review and meta-analysis, the prevalence of depression among patients with T2D was estimated to be 28 % worldwide and 24 % in Europe [36]. Our study found a slightly higher prevalence when evaluations were based on self-reports (30 %) but its diagnosis-based frequency was still only 22 %. One explanation for this might be that depression diagnoses had not been properly recorded on the patients’ registers. On the other hand, it might be that depression was underdiagnosed in our primary care population. A Spanish multicentre primary care study observed that 12 % of T2D patients had undiagnosed depression [37], and other studies from different clinics (not only primary care) have yielded similar percentages (17 and 18 %) [38, 39]. Due to the increased mortality and cardiac event risks related to comorbid depression [40], and on the other hand, the favourable treatment outcomes of depression from glycaemic control [41], screening and treating depression among patients with T2D should definitely be a crucial part of T2D treatment.

Multimorbidity in general and T2D-related (concordant) diseases were associated with achievement of the HbA1c treatment target among the patients with T2D, but we observed no association with having only discordant diseases. Lin et al. [42] explored over 160,000 patients with T2D and observed that those with concordant diseases only or in combination with discordant disease(s) were more likely to achieve the HbA1c treatment target than those with T2D only. Similar findings have been reported in large studies assessing diabetes without separating type 1 and 2 [16, 19]. Furthermore, Magnan et al. [16] and Woodard et al. [19] showed significant associations between concordant diseases and the attainment of goals for cholesterol/LDL, but their findings with respect to BP target achievement were contradictory. According to our cross-sectional results, concordant diseases alone play no role in these outcomes among patients with T2D, but co-existence with discordant disease(s) seems to be relevant with respect to meeting the LDL target. The smaller significance of having discordant diseases only, in comparison to concordant diseases, for T2D treatment goal achievement has also been observed earlier in some diabetic populations [16, 18], although not in all [19]. Perhaps patients with concordant diseases are treated more efficiently and are more adherent to medication [10] than those with T2D or non-T2D-related diseases only, as they have other essential conditions that need to be at an optimal level to reduce the risks of T2D complications. However, this does not explain the smaller significance of concordant disease burden in the achievement of sBP treatment goals among patients with T2D, and further investigations are warranted.

The main strengths of the current study are its large primary care study population of patients with T2D, its real-world setting, and its reliable register-based data with no recall bias. Furthermore, the services of the municipal health care system of the city, like those of Finland in general, are low or moderate in cost and therefore the wealth of an individual is unlikely to hinder visits to physicians. To our knowledge, this is the first study to evaluate multimorbidity and its subgroups’ relations to the achievement of T2D treatment targets among a large set of Finnish patients with T2D. However, some limitations must also be acknowledged. One clear limitation of our study is related to the structure of the register data, which means that some diseases may be under-reported/-diagnosed, such as depression, the prevalence of which was lower than we expected (6 %; data not shown). Due to the low rate of obesity, we also calculated and reported BMI. With respect to other limitations, the cross-sectional nature of the present study prevented us from making conclusions about the cause-and-effect relationships between multimorbidity and achievement of T2D treatment goals. As we used a real-world clinical dataset, we had no data on lifestyle factors (except BMI), which can also be seen as a limitation. Some working-age patients with T2D may be treated by occupational health services only, and would thus not have been part of the present data. The data on prescribed medications were collected 2 years before and after the last clinical values of HbA1c, LDL and sBP, which may have led to the exclusion of some prescriptions. However, the defined timeline was considered to describe the best estimate of the current use of medications, as unfortunately, exact data were not available.

## Conclusions

Multimorbidity is highly prevalent among patients with T2D. Diseases considered to be cardiovascular risk factors (hypertension and hyperlipidaemia) and MS diseases were emphasised in multimorbidity patterns. Multimorbidity in general and T2D-related concordant diseases alone or in combination with non-T2D-related discordant diseases were associated with the achievement of the HbA1c treatment goal, in comparison to T2D only. A combination of both concordant and discordant diseases was also related to meeting the LDL treatment goal, in comparison to T2D only. Clinicians working in primary care clinics should focus not only on patients with possible multimorbidity and morbidity including concordant diseases, but also on patients with T2D only, to provide support with their disease burden and maintain the targets of HbA1c and LDL treatment. In the future, the possible predictive role of multimorbidity in the achievement of treatment targets for T2D should be explored in a longitudinal setting. Similarly, attention should be paid to investigating different putative ways to improve the achievement of treatment targets among patients with T2D only.

## Data Availability

The data are not publicly available, to protect patients’ confidentiality. All relevant data are presented in the article.

## Abbreviations

T2D: Type 2 diabetes
HbA1c: Glycosylated haemoglobin A1c
LDL: Low-density lipoprotein
BP: Blood pressure
sBP: Systolic blood pressure
OR: Odds ratio
CI: Confidence interval
ICD-10: 10th revision of the World Health Organization’s International Classification of Disease
COPD: Chronic obstructive pulmonary disease
MS: Musculoskeletal
BMI: Body mass index
ATC: Anatomical therapeutic chemical
eGFR: Estimated glomerular filtration rate
SD: Standard deviation
